# Morphological Variety in *Distoseptispora* and Introduction of Six Novel Species

**DOI:** 10.3390/jof7110945

**Published:** 2021-11-09

**Authors:** Jing Yang, Ling-Ling Liu, E. B. Gareth Jones, Wen-Li Li, Kevin D. Hyde, Zuo-Yi Liu

**Affiliations:** 1 Guizhou Institute of Soil and Fertilizer, Guizhou Academy of Agricultural Sciences, Guiyang 550006, China; yangjing5633@gmail.com; 2 Center of Excellence in Fungal Research, Mae Fah Luang University, Chiang Rai 57100, Thailand; kdhyde3@gmail.com; 3 School of Life Science and Technology, University of Electronic Science and Technology of China, Chengdu 611731, China; wendy316@tom.com; 4 Guizhou Key Laboratory of Agricultural Biotechnology, Guizhou Academy of Agricultural Sciences, Guiyang 550006, China; gzlzy8558@gmail.com; 5 Department of Entomology and Plant Pathology, Faculty of Agriculture, Chiang Mai University, Chiang Mai 50200, Thailand; torperadgj@gmail.com; 6 Department of Botany and Microbiology, College of Science, King Saud University, Riyadh 11451, Saudi Arabia

**Keywords:** new taxa, generic delimitation, molecular phylogeny, multi-gene, sporidesmium-like taxa

## Abstract

*Distoseptispora* is one of the sporidesmium-like taxa with great variation in asexual morphology and delineation of species. Phylogenetic analyses of four gene regions LSU, ITS, *TEF1α*, and *RPB2* revealed the placement of several sporidesmium-like species in *Distoseptispora* (*Distoseptisporaceae*, *Distoseptisporales*, *Sordariomycetes*), collected on submerged decaying twigs from streams in China and Thailand. Based on morphological examination and molecular DNA data, six new species, *Distoseptispora amniculi*, *D. atroviridis*, *D. effusa*, *D. fusiformis*, *D. hyalina*, and *D. verrucosa*, are proposed. Among them, *D.* *hyalina* is the first sexual morph confirmed in the genus. A new geographical record is reported for *D. lignicola* in China. Conidial length proved to be of less taxonomic significance for some *Distoseptispora* species, whereas the type of conidial septa is informative at species level.

## 1. Introduction

The dematiaceous sporidesmium-like hyphomycetes are common saprobes on decaying wood from terrestrial and freshwater habitats and distributed worldwide. They are characterized by holoblastic phragmoconidia produced on macronematous, proliferating or non-proliferating conidiophores or reduced to conidiogenous cells [1,2]. Considering the taxonomic value of euseptate and distoseptate conidia, absence or presence of conidiophores, conidiophore proliferation, and the shape of conidiogenous cells, several genera segregated from *Sporidesmium sensu lato* and allied genera were introduced, e.g., *Ellisembia*, *Imicles*, *Penzigomyces*, *Polydesmus*, *Repetophragma*, *Sporidesmiella*, and *Stanjehughesia* [1,3,4,5,6]. Later, with the incorporation of molecular data in phylogenetic studies, *Sporidesmium sensu lato* and morphologically similar genera were revealed to be polyphyletic mainly within *Dothideomycetes* and *Sordariomycetes*. Many of the morphological characters used to delimit the genera did not appear phylogenetically significant [7,8,9,10,11].

*Distoseptispora* is one of the sporidesmium-like genera introduced by Su et al. [9] with the type species *D. fluminicola* and the second species *D. aquatica* having long, cylindrical, distoseptate conidia. Yang et al. [10] emended the generic concept of *Distoseptispora* based on the morphological features of *D. guttulata* and *D. suoluoensis*, namely taxa with conspicuously longer conidiophores elongating percurrently and obclavate, rostrate, euseptate conidia. The generic circumscription was later expanded since more species with new morphological traits were described in the genus, such as *D. palmarum* [12] having polyblastic conidiogenous cells, *D. appendiculata* [13] and *D. hydei* [14] characterized by a mucilaginous conidial sheath, and *D. martinii* (Basionym: *Acrodictys martinii*) with muriform, transverse conidia [15]. Members in *Distoseptispora* are commonly found in freshwater habitats, and many new species have been discovered from China and Thailand in recent years [10,11,14,16,17,18,19,20,21,22,23,24,25]. Currently, more than 30 species are accepted in the genus but without a known sexual morph.

It is challenging to classify *Distoseptispora* species based on morphology alone because of the high morphological similarity to *Sporidesmium* and *Ellisembia* with euseptate and distoseptate conidia, respectively. *Distoseptispora* forms a monophyletic clade that is distinct from other sporidesmium-like taxa [9,10,13]. The genus was the only member belonging to *Distoseptisporaceae* and together with the monotypic *Aquapteridosporaceae* in *Distoseptisporales* [26,27].

During our survey of the taxonomy and diversity of freshwater fungi along a north–south gradient in the Asian/Australasian region [28], several sporidesmium-like taxa were collected from freshwater streams in China and Thailand. Using multi-gene loci of LSU, ITS, *TEF1α*, and *RPB2* gene regions, the systematic placement of these collections revealed several *Distoseptispora* species. Based on the morphology and molecular evidence, we introduce six novel species in *Distoseptispora* including a sexual morph for *D. hyalina* and a new geographical record for *D. lignicola* in China. An updated generic description and backbone tree of *Distoseptispora* is provided and its generic delimitation discussed.

## 2. Materials and Methods

### 2.1. Collection and Examination of Specimens

Specimens of submerged decaying twigs were collected from streams in China and Thailand. Samples were taken to the laboratory in zip-lock plastic bags and incubated in plastic boxes lined with moistened tissue at room temperature for one week. Motic SMZ 168 Series (Motic, Xiamen, China) and Nikon SMZ-171 (Nikon, Tokyo, Japan) dissecting microscopes were used to observe the fungal colonies and fruiting bodies. Fungal structures were examined and photographed using a Nikon ECLIPSE 80i (Nikon, Tokyo, Japan) compound microscope fitted with a Canon 600D/70D (Canon, Tokyo, Japan) digital camera. Single spore isolations were made onto freshwater agar (WA) or potato dextrose agar (PDA) and germinated spores were transferred onto malt extract agar (MEA) or PDA following the method in Luo et al. [17]. Tarosoft Image Frame Work (Tarosoft, Nontha Buri, Thailand) was used for measurement and images used for figures were processed with Adobe Photoshop CC 2019 (Adobe Systems, San Jose, CA, USA) software. Herbarium specimens (dry wood with fungal material) were deposited in the herbarium of Mae Fah Luang University (MFLU), Chiang Rai, Thailand and herbarium of Cryptogams, Kunming Institute of Botany Academia Sinica (HKAS), Kunming, China. Axenic cultures were deposited in Mae Fah Luang University Culture Collection (MFLUCC) and Guizhou Culture Collection (GZCC). Facesoffungi and Index Fungorum numbers were registered as outlined in Jayasiri et al. [29] and Index Fungorum [30].

### 2.2. DNA Extraction, PCR Amplification, and Sequencing

Germinated spores were grown on MEA/PDA medium at 25 °C for one month. Fungal mycelium was scraped off using a sterilized scalpel and transferred to a 1.5 mL microcentrifuge tube for genomic DNA extraction. A Ezup Column Fungi Genomic DNA Purification Kit (Sangon Biotech, Shanghai, China) was used to extract DNA following the manufacturer’s instructions. DNA amplification was performed by polymerase chain reaction (PCR). LSU, SSU, ITS, *TEF1α*, and *RPB2* gene regions were amplified using the primer pairs LR0R/LR5, NS1/NS4, ITS5/ITS4, 983F/2218R, and fRPB2-5F/fRPB2-7cR [31,32,33,34]. The amplifications were performed in a 25 μL reaction volume containing 9.5 μL ddH_2_O, 12.5 μL 2 × Taq PCR Master Mix with blue dye (Sangon Biotech, China), 1 μL of DNA template, and 1 μL of each primer (10 μM). The amplification condition for LSU, SSU, ITS, and *TEF1α* consisted of initial denaturation at 94 °C for 3 min, followed by 40 cycles of 45 s at 94 °C, 50 s at 56 °C, and 1 min at 72 °C, and a final extension period of 10 min at 72 °C. The amplification condition for *RPB2* gene consisted of initial denaturation at 95 °C for 5 min, followed by 37 cycles of 15 s at 95 °C, 50 s at 56 °C, and 2 min at 72 °C, final extension period of 10 min at 72 °C. Purification and sequencing of PCR products were carried out by Shanghai Sangon Biological Engineering Technology and Services Co., Shanghai, China.

### 2.3. Phylogenetic Analyses

The ex-type and additional strains of *Distoseptisporaceae* species and related families (*Acrodictyaceae*, *Aquapteridosporaceae*, *Bullimycetaceae*, *Cancellidiaceae*, *Papulosaceae*, and *Pseudostanjehughesiaceae*) were selected in the phylogenetic analyses (Table 1). Four gene regions LSU, ITS, *TEF1α*, and *RPB2* were used for the multi-gene analyses. Sequences were optimized manually to allow maximum alignment and maximum sequence similarity. The sequences were aligned using the online multiple alignment program MAFFT v.7 (Available online: http://mafft.cbrc.jp/alignment/server/ (accessed on 3 August 2021)) [35]. The alignments were checked visually and improved manually where necessary.

Maximum likelihood (ML), Bayesian inference (BI), and maximum parsimony (MP) analyses were employed to assess phylogenetic relationships. ML and BI analyses were performed through the CIPRES science Gateway V. 3.3 [36]. ML analyses were conducted with RAxML-HPC v. 8.2.12 [37] using a GTRGAMMA approximation with rapid bootstrap analysis followed by 1000 bootstrap replicates. For the BI approach, MrModeltest2 v. 2.3 [38] was used to infer the appropriate substitution model that would best fit the model of DNA evolution for the combined dataset. GTR + G + I substitution model was selected for LSU, ITS, *TEF1α*, and *RPB2* partitions. BI analyses were performed in a likelihood framework as implemented in MrBayes 3.2.6 [39]. Six simultaneous Markov chains were run until the average standard deviation of split frequencies was below 0.01, with trees saved every 1000 generations. The first 25% of saved trees, representing the burn-in phase of the analysis, were discarded. The remaining trees were used for calculating posterior probabilities of recovered branches [40]. MP analyses were conducted with PAUP v. 4.0a167 [41]. A heuristic search was performed with the stepwise-addition option with 1000 random taxon addition replicates and tree bisection and reconnection branch swapping. All characters were unordered and of equal weight and gaps were treated as missing data. Maxtrees were unlimited, branches of zero length were collapsed, and all multiple, equally parsimonious trees were saved. Clade stability was assessed using a bootstrap analysis with 1000 replicates, each with 10 replicates of random stepwise addition of taxa [42].

The resulting trees were printed with FigTree v. 1.4.4 and the layout was created in Adobe Illustrator 2019 (Adobe Systems, San Jose, CA, USA). Sequences generated in this study were deposited in GenBank (Table 1).

## 3. Phylogenetic Results

**Figure 1:**
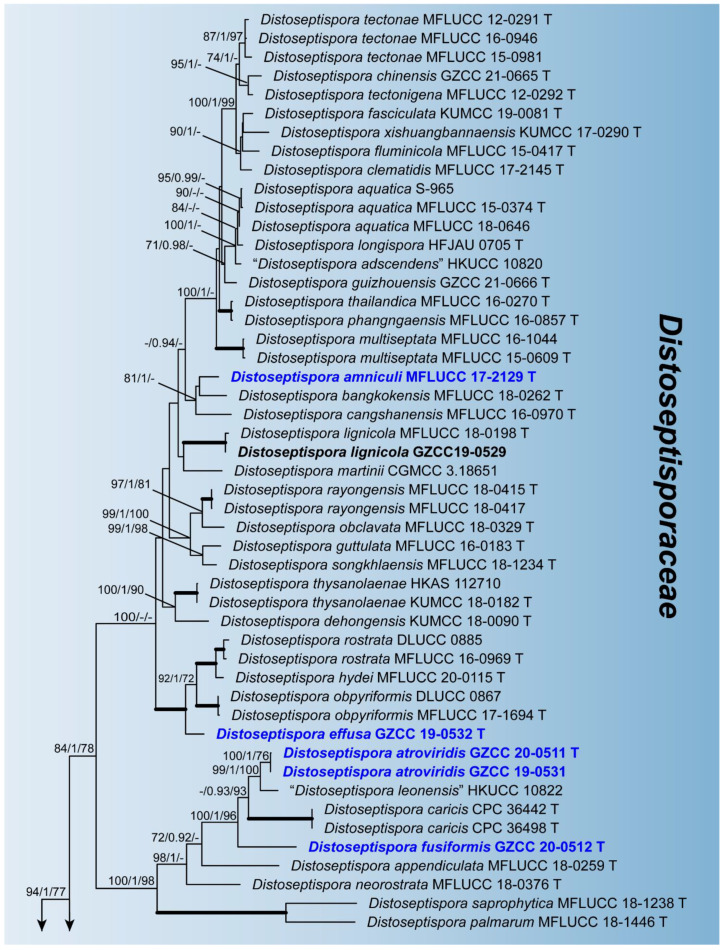

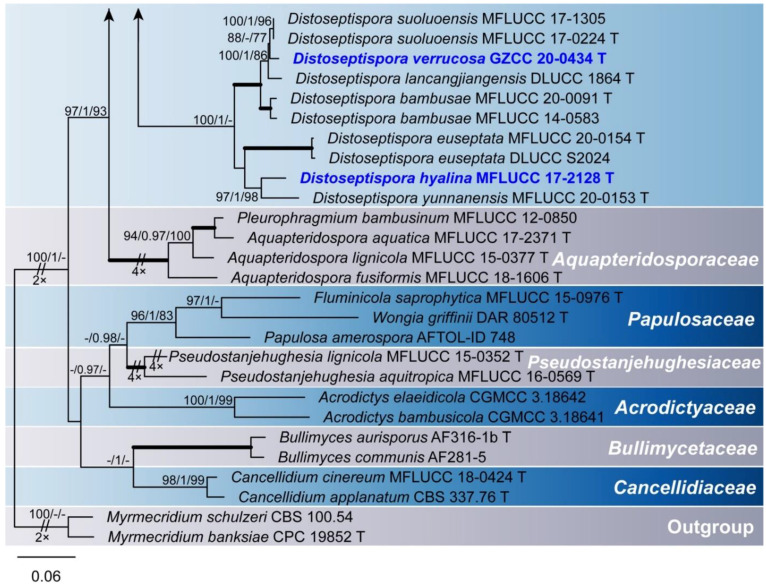
Maximum likelihood majority rule consensus tree for *Distoseptisporaceae* and related families using LSU, ITS, *TEF1α*, and *RPB2* sequence data. Bootstrap support values for maximum likelihood (ML) and maximum parsimony (MP) greater than 70% and Bayesian posterior probabilities greater than 0.90 are indicated above branches as ML BS/PP/MP BS. The scale bar represents the expected number of changes per site. The tree is rooted with *Myrmecridium schulzeri* (CBS 100.54) and *Myrmecridium banksiae* (CPC 19852). Ex-type strains are indicated with T. The new collections are in bold with new taxa in blue. Branches with 100% ML BS, 1.0 PP, and 100% MP BS are thickened. Families are indicated as colored blocks.

Phylogenetic relationships of seven *Distoseptispora* species were assessed in the combined analysis using four gene regions of 76 strains representing 61 species in *Distoseptisporaceae* and related families (*Acrodictyaceae*, *Aquapteridosporaceae*, *Bullimycetaceae*, *Cancellidiaceae*, *Papulosaceae*, and *Pseudostanjehughesiaceae*). The analyzed alignment consisted of combined LSU (1–858 bp), ITS (859–1522 bp), *TEF1α* (1523–2432 bp), and *RPB2* (2433–3490 bp) sequence data including gaps. *Myrmecridium schulzeri* (CBS 100.54) and *Myrmecridium banksiae* (CPC 19852) served as outgroup taxa. The best scoring RAxML tree is shown in Figure 1. The analyzed ML, MP, and Bayesian trees were similar in topology and did not conflict significantly. *Distoseptispora* was resolved as a monophyletic clade. Our eight strains nested within the genus representing seven species. *Distoseptispora amniculi* (MFLUCC 17-2129) clustered as sister taxon to *D. bangkokensis* (MFLUCC 18-0262) but with weak support. *Distoseptispora effusa* (GZCC 19-0532) formed a distinct clade sister to the clade containing *D. hydei* (MFLUCC 20-0115), *D. rostrata* (MFLUCC 16-0969 and DLUCC 0885), and *D. obpyriformis* (MFLUCC 17-1694 and DLUCC 0867) with strong statistical support (100% ML BS/1.0 PP/100% MP BS). *Distoseptispora atroviridis* (GZCC 20-0511 and GZCC 19-0531), based on two strains, grouped with “*D. leonensis*” (HKUCC 10822) and was close to *D. fusiformis* (GZCC 20-0512). *Distoseptispora verrucosa* (GZCC 20-0434) grouped with *D. suoluoensis* (MFLUCC 17-1305 and MFLUCC 17-0224), *D. lancangjiangensis* (DLUCC 1864), and *D. bambusae* (MFLUCC 20-0091 and MFLUCC 14-0583), in a sister group of *D. euseptata* (MFLUCC 20-0154 and DLUCC S2024), *D. hyalina* (MFLUCC 17-2128), and *D. yunnanensis* (MFLUCC 20-0153), forming a statistically well supported clade by ML and BI analyses. Members of this clade have similar conidial morphology except *D. hyalina* in sexual stage. The strain GZCC 19-0529 positioned sister to the ex-type strain (MFLUCC 18-0198) of *D. lignicola* with identical LSU sequences and three base pair differences in ITS sequences, and therefore is identified as *D. lignicola* based on the morphology and molecular evidence.

## 4. Taxonomy

*Distoseptispora* K.D. Hyde, McKenzie, and Maharachch., Fungal Diversity 80: 402 (2016).

Index Fungorum number: IF551833; Facesoffungi number: FoF01755.

**Sexual morph:** Ascomata solitary or gregarious, immersed to semi-immersed, perithecial, subglobose to ellipsoidal, dark brown, ostiolate, with a short neck. Ostiole periphysate. Ascomatal wall coriaceous, 2-layered, outer layer consisting of multi-rows of brown, thick-walled, polyhedral cells of textura angularis, inner layer comprising multi-rows of pale brown to hyaline, thin-walled, elongated cells of textura angularis or taxtura prismatica. Paraphyses sparse, persistent, septate, hyaline, tapering towards the apex, constricted at the septa. Asci unitunicate, 8-spored, cylindrical, pedicellate, obtuse at the apex, apex with a non-amyloid apical annulus. Ascospores overlapping, uniseriate, fusiform, hyaline, 0–3-septate, smooth-walled, guttulate, thin-walled, with a mucilaginous sheath. **Asexual morph:** Hyphomycetous. Colonies effuse, hairy, velvety, olivaceous to dark brown. Mycelium mostly immersed, composed of branched, septate, smooth, pale brown hyphae. Conidiophores macronematous, mononematous, erect, single or in groups or fasciculate, septate, unbranched, straight or slightly flexuous, smooth, olivaceous to brown, cylindrical, rounded or truncate at the apex, sometimes elongating percurrently, rarely reduced to conidiogenous cells. Conidiogenous cells mostly monoblastic, sometimes polyblastic, integrated, determinate, terminal, cylindrical or clavate with flared apex. Conidia acrogenous, solitary, cylindrical or obclavate, rostrate, ellipsoidal, obovoid to fusiform, subhyaline, olivaceous, dark green, brown or yellowish-brown to reddish-brown, euseptate or distoseptate, rarely muriform, truncate at base, sometimes indeterminate in length or producing a secondary conidium, septal pore and mucilaginous sheath present or absent.

Type species—*Distoseptispora fluminicola* McKenzie, H.Y. Su, Z.L. Luo, and K.D. Hyde

Notes: Hyde et al. [11] provided the family description for the monotypic *Distoseptisporaceae*. The diagnosis of the sexual morph in the family was based on *Miyoshiella triseptata*, which was associated with “*Distospetispora adscendens*” (as *Ellisembia adscendens*) in the same collection [7,43]. However, neither the cultural study nor molecular data has proved their connection. We prefer to treat *Miyoshiella triseptata* as a possible sexual morph of sporidesmium-like taxa. The sexual description here is based on *Distoseptispora hyalina*. *Distoseptispora martinii* is unique in the genus by transverse ellipsoid or subglobose, muriform conidia [15]. Additional collections and further molecular evidence are needed to confirm its taxonomy.

**Figure 2:**
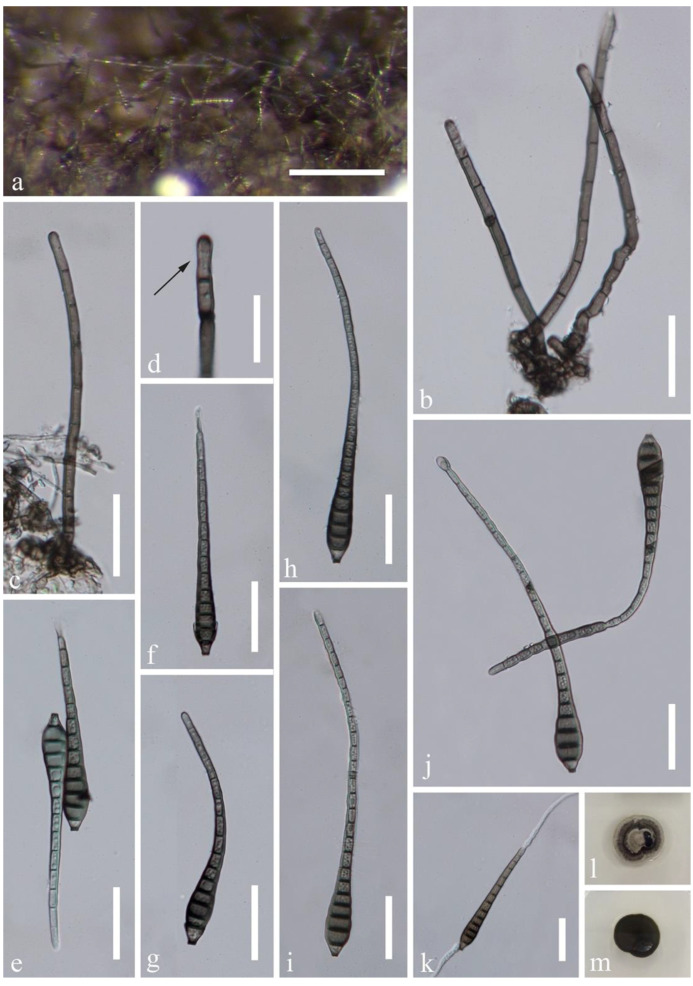
*Distoseptispora amniculi* (MFLU 21-0138, holotype). (**a**) Colonies on wood. (**b**,**c**) Conidiophores. (**d**) Conidiogenous cell. (**e**–**j**) Conidia. (**k**) Germinated conidium. (**l**,**m**) Colony on PDA, l from above, m from below. Scale bars: (**a**) 200 µm, (**b**,**c**,**e**–**k**) 30 µm, (**d**) 20 µm.

*Distoseptispora amniculi* J. Yang and K.D. Hyde, sp. nov., Figure 2.

Index Fungorum number: IF558670; Facesoffungi number: FoF10250.

Etymology: referring to the collecting site of a small stream.

Holotype: MFLU 21-0138.

Saprobic on submerged decaying wood in a freshwater habitat. **Asexual morph:** Colonies on wood effuse, hairy, dark brown, scattered or in small groups, glistening, usually retiform. Mycelium partly immersed, partly superficial, composed of septate, smooth-walled, pale brown to hyaline hyphae. Conidiophores macronematous, mononematous, erect, solitary or caespitose, straight or flexuous, cylindrical, rounded at the apex, smooth-walled, septate, unbranched, grayish brown, 90–180 × 3–4.5 µm ($\bar{x}$ = 125 × 4 µm, n = 20). Conidiogenous cells monoblastic, integrated, terminal, determinate, cylindrical, pale brown, rounded and darkened at the apex, sometimes elongating percurrently. Conidia acrogenous, obclavate, rostrate, olivaceous brown, grayish brown or mid brown, paler towards the apex, (7–)12–24-septate, 85–167 × 9–11.8 µm ($\bar{x}$ = 120 × 10 µm, n = 20), smooth-walled, truncate and darkened at the base, sometimes with a secondary conidium. **Sexual morph:** undetermined.

Culture characteristics: conidia germinating on PDA within 24 h and germ tubes produced from both ends. Colonies growing on PDA slow growing, reaching 10–15 mm in a month at 25 °C in natural light, circular, with dense, gray mycelium in the middle, darker of the inner ring, with sparser, white mycelium of the outer ring on the surface, in reverse dark brown to black with smooth margin.

Material examined: Thailand, Trat Province, Amphoe Ko Chang, 12°7.98′ N, 102°37.98′ E, on decaying wood submerged in a freshwater stream, 27 April 2017, YZ Lu, YJT 26-2 (MFLU 21-0138, **holotype**); ex-type living culture MFLUCC 17-2129; additional sequence, SSU: MZ868766.

Notes: *Distoseptispora amniculi* is similar to *D. neorostrata* and *D. rostrata* in the relative long conidiophores (more than 80 µm long) and obclavate, rostrate, distoseptate conidia. *Distoseptispora neorostrata* [13] can be distinguished from the present species in having truncate apex to the conidiophores and dark and wider conidia (109–147 × 13–15 µm); *D. rostrata* [17] has olivaceous to pale brown conidia that are 115–155 × 9–11 µm. Conidiophores of *D. amniculi* are longer than that in *D. neorostrata* (90–180 µm vs. 93–117 µm) and *D. rostrata* (90–180 µm vs. 82–126 μm). In the multi-gene phylogenetic tree (Figure 1), *D. amniculi* clustered with *D. bangkokensis*. They have distoseptate conidia and rounded apex of conidiophores. *Distoseptispora amniculi* is well distinguishable from *D. bangkokensis* [44] by longer conidiophores (90–180 µm vs. 37–55 µm) and shorter conidia (85–167 µm vs. 400–568 µm). Comparison of the LSU, ITS, and SSU sequences of *D. amniculi* and *D. bangkokensis* showed 99.75% (798/800bp), 92.91% (485/522bp including 11bp of gaps), and 99.44% (893/898bp) sequence identity, respectively.

**Figure 3:**
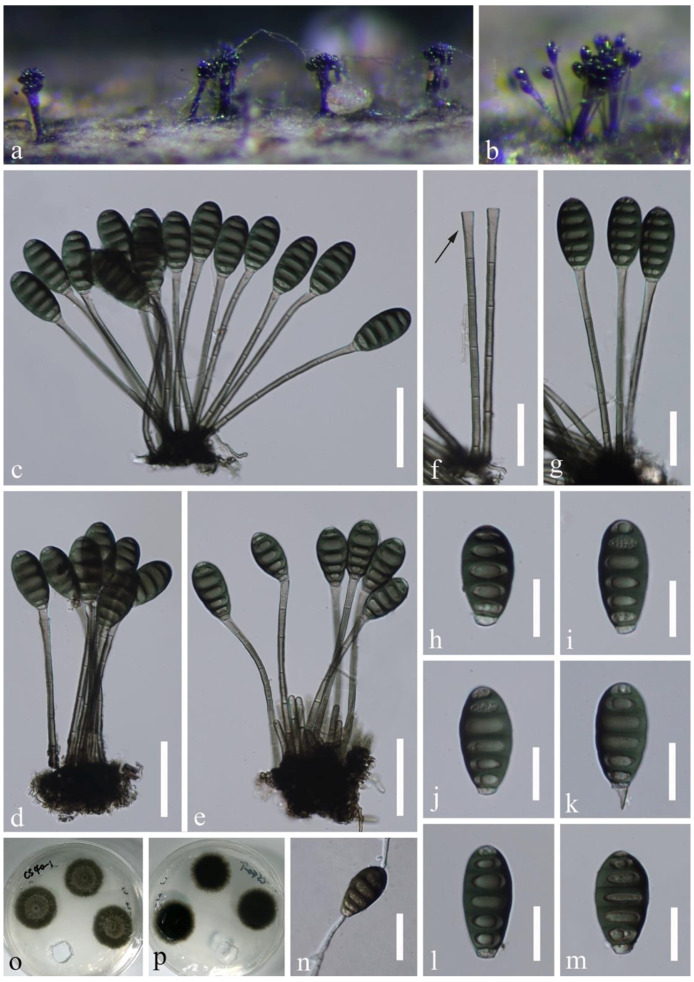
*Distoseptispora atroviridis* (HKAS 112616, holotype). (**a**,**b**) Colonies on woody substrates. (**c**–**e**,**g**) Fasciculate conidiophores with conidia. (**f**) Conidiophores and conidiogenous cells. (**h**–**m**) Conidia. (**n**) Germinated conidium. (**o**,**p**) Culture, (**o**) from above, (**p**) from below. Scale bars: (**c**–**e**) 50 μm, (**f**,**g**,**n**) 30 μm, (**h**–**m**) 20 μm.

*Distoseptispora atroviridis* J. Yang and K.D. Hyde, sp. nov., Figure 3.

Index Fungorum number: IF558671; Facesoffungi number: FoF10252.

Etymology: referring to the dark green conidia.

Holotype: HKAS 112616.

Saprobic on submerged decaying wood in a freshwater habitat. **Asexual morph:** Colonies on wood effuse, dark brown to black, scattered or in small groups, glistening. Mycelium mostly immersed, composed of septate, smooth-walled, brown to hyaline hyphae. Conidiophores macronematous, fasciculate, loosely compact, erect, straight or slightly flexuous, cylindrical, wider and truncate at the apex, smooth-walled, septate, unbranched, pale grayish brown, slightly paler at the apical cell, 100–167 × 2.7–4 µm ($\bar{x}$ = 124.5 × 3.5 µm, n = 30), 4.7–6.9 µm wide at the apex. Conidiogenous cells monoblastic, integrated, terminal, determinate, sometimes elongating percurrently, flared, pale grayish brown. Conidia acrogenous, solitary, ellipsoidal to obovoid, dark green, subhyaline at the basal cell, 6-septate, 31–43 × 13–20 µm ($\bar{x}$ = 39 × 18 µm, n = 40), smooth-walled, guttulate, truncate at the base, sometimes released with part of conidiogenous cells. **Sexual morph:** Undetermined.

Culture characteristics: conidia germinating on PDA within 24 h and swollen germ tubes produced from both ends. Colonies growing on PDA reaching 5–10 mm in two weeks at 25 °C in the dark, with dense, velvety, dark green mycelium on the surface; in reverse dark green with entire margin.

Material examined: China, Guizhou Province, Chishui City, Sidonggou Waterfall, 25°27.38′ N, 107°39.85′ E, on submerged decaying twig in a freshwater stream, 11 July 2019, J Yang, CS 40-1 (HKAS 112616, **holotype**); ex-type living culture GZCC 20-0511; ibid, near 28°25′ N, 106°0′ E, at an altitude of 500 m, on submerged decaying wood in a freshwater stream, 16 July 2019, LL Liu, CS 1-4-2 (GZAAS 20-0426, **paratype**); living culture GZCC 19-0531 (additional sequence, SSU: MW134695).

Notes: *Distoseptispora atroviridis* is well distinguishable among other species of the genus by fasciculate, synnematous-like conidiophores, flared conidiogenous cells and ellipsoidal to obovoid, dark green, 6-septate conidia with paler to subhyaline basal cell. *Distoseptispora atroviridis* resembles some *Phragmocephala* species, such as *P. atra*, *P. elliptica*, *P. hughesii*, and *P. garethjonesii*, in having loosely to compactly fasciculate conidiophores aggregated at the base, clavate, flared conidiogenous cells sometimes elongating percurrently and ellipsoidal to obovoid conidia that secedes rhexolyticly [6,45]. However, *Phragmocephala* species differ from *D. atroviridis* by brown conidia with thickened and darkened bands. Molecular analyses revealed the placement of *Phragmocephala* in *Melanommataceae* (*Pleosporales*, *Dothideomycetes*) distinct from *D. atroviridis* [45,46]. In our phylogenetic tree, *D. atroviridis* (GZCC 20-0511 and GZCC 19-0531) was sister to “*Distoseptispora leonensis*” HKUCC10822 (99% ML BS/1.0 PP/100% MP BS, Figure 1), but they are distinguishable in morphology. “*Distoseptispora leonensis*” was characterized by solitary, mid to dark brown conidiophores with up to three successive proliferations [47] while *D. atroviridis* has fasciculate, loosely compact, longer but narrower conidiophores (100–167 × 2.7–4 µm vs. 70–120 × 4.5–8 µm) rarely percurrently proliferating. Conidiogenous cells are cylindrical and slightly narrower at the apex in “*D. leonensis*” [47] while those are flared with wider apex in *D. atroviridis*. The former species has fusiform or rostrate, mid to dark brown, 9–17-septate conidia with tapering apical cells [47] while the latter has ellipsoidal to obovoid, dark green, 6-septate, smaller conidia (31–43 × 13–20 µm vs. 45–90 × 15–18 µm).

**Figure 4:**
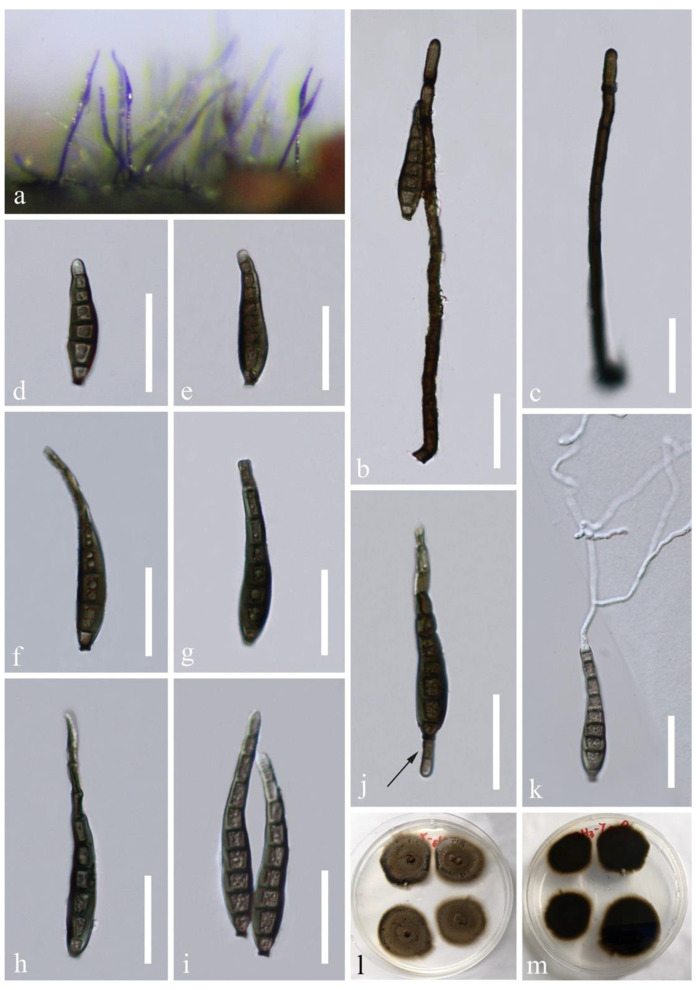
*Distoseptispora effusa* (GZAAS 20-0427, holotype). (**a**) Colonies on natural substrate. (**b**,**c**) Conidiophores. (**d**–**i**) Conidia. (**j**) Conidiogenous cell with a conidium. (**k**) Germinated conidium. (**l**,**m**) Culture, l from above, m from below. Scale bars: (**b**–**k**) 50 μm.

*Distoseptispora effusa* L.L. Liu and Z.Y. Liu, sp. nov., Figure 4.

Index Fungorum number: IF558406; Facesoffungi number: FoF09863.

Etymology: referring to the effuse colonies.

Holotype: GZAAS 20-0427.

Saprobic on decaying wood in a freshwater habitat. **Asexual morph:** Colonies on natural substrates superficial, effuse, dark brown to black, hairy, velvety. Mycelium mostly immersed, consisting of branched, hyaline to pale brown, smooth, septate hyphae. Conidiophores macronematous, mononematous, erect, solitary or in small groups, cylindrical, dark brown, 5–14-septate, straight or slightly curved, smooth, 72–171 × 5–6.5 μm ($\bar{x}$ = 104.5 × 5.5 μm, n = 15), rounded at the apex. Conidiogenous cells monoblastic, integrated, terminal, determinate, cylindrical, brown, sometimes elongating percurrently, darkened at the rounded apex and percurrent loci. Conidia acrogenous, solitary, obclavate, rostrate, smooth-walled, olivaceous brown to dark brown, sometimes slightly paler at the apex, straight or slightly curved, 4–9-distoseptate, 35.5–113 × 7–12.5 μm ($\bar{x}\;$ = 71 × 10 μm, n = 20), truncate and darkened at the base. **Sexual morph:** Undetermined.

Cultural characteristics: conidia germinated on WA within 24 h and germ tube produced from the apex. Colonies on PDA reaching 15–20 mm diam. after 2 weeks at 25 °C in dark, circular, with fluffy, dense, dark olivaceous brown aerial mycelium on the surface; in reverse dark brown with entire margin.

Material examined: China, Guizhou Province, Chishui City, Chishui river basin, near 28°25′ N, 106°0′ E, at an altitude of 500 m, on submerged decaying wood in a freshwater stream, July 2019, LL Liu, CS 3-7 (GZAAS 20-0427, **holotype**); ex-type living culture GZCC 19-0532; additional sequences, SSU: MW134696.

Notes: Phylogenetically, *Distoseptispora effusa* (GZCC 19-0532) nested within *Distoseptispora* and formed a distinct clade sister to the clade containing *D. rostrata*, *D. hydei*, and *D. obpyriformis* (Figure 1). Morphologically, *D. effusa* is similar to *D. rostrata* [17] with percurrently elongate conidiophores and obclavate, distoseptate conidia but differs by longer conidiophores (72–171 μm vs. 82–126 μm) and shorter conidia (35.5–113 μm vs. 115–155 μm). *Distoseptispora hydei* [14] is distinguished by fusiform to obpyriform, light olivaceous to brown conidia. *Distoseptispora obpyriformis* [17] has obclavate to obpyriform, olivaceous to dark brown conidia that are shorter but wider than *D. effusa* (53–71 × 12–16 μm vs. 35.5–113 × 7–12.5 μm).

**Figure 5:**
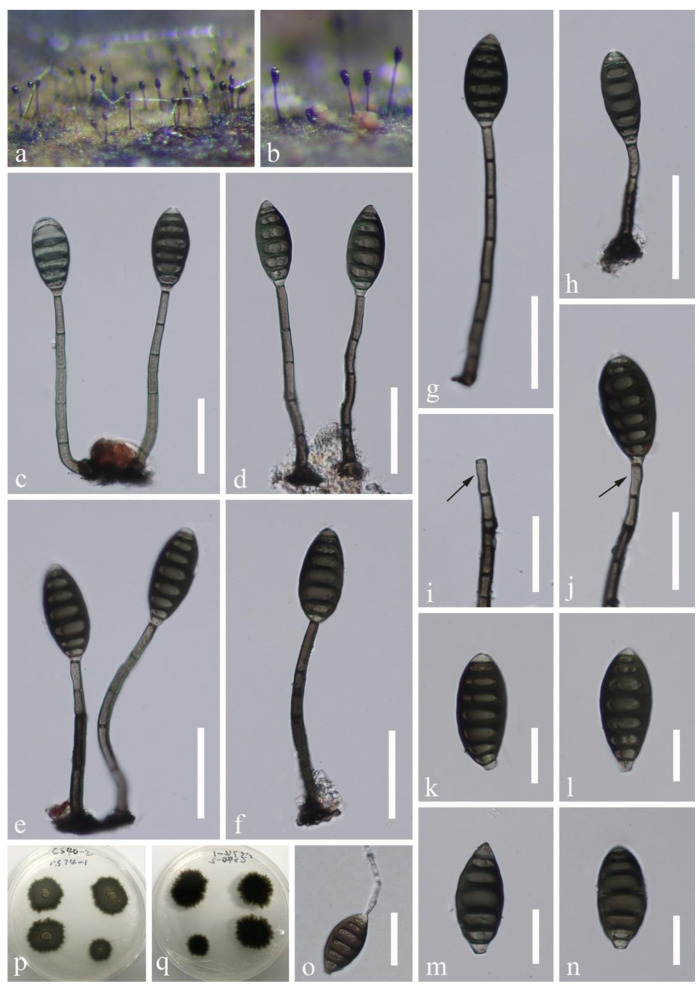
*Distoseptispora fusiformis* (HKAS 112617, holotype). (**a**,**b**) Colonies on woody substrates. (**c**–**h**) Conidiophores with conidia. (**i**,**j**) Conidiogenous cells. (**k**–**n**) Conidia. (**o**) Germinated conidium. (**p**,**q**) Culture, p from above, q from below. Scale bars: (**c**–**h**) 40 μm, (**i**,**j**,**o**) 30 μm, (**k**–**n**) 20 μm.

*Distoseptispora fusiformis* J. Yang and K.D. Hyde, sp. nov., Figure 5.

Index Fungorum number: IF558672; Facesoffungi number: FoF10253.

Etymology: referring to the fusiform conidia.

Holotype: HKAS 112617.

Saprobic on submerged decaying wood in a freshwater habitat. **Asexual morph:** Colonies on wood effuse, hairy, dark brown to black, scattered or in small groups, glistening. Mycelium mostly immersed, composed of septate, smooth-walled, brown to hyaline hyphae. Conidiophores macronematous, mononematous, erect, straight or slightly flexuous, cylindrical, slightly tapering towards the apex, smooth-walled, septate, unbranched, pale to dark brown, slightly paler at the apical cells, 40–110 × 3.5–5.8 µm ($\bar{x}$ = 86 × 4.6 µm, n = 20). Conidiogenous cells monoblastic, integrated, terminal, determinate, sometimes elongating percurrently, cylindrical, brown. Conidia acrogenous, solitary, ellipsoidal to fusiform, dark olivaceous brown to dark brown, pale brown at both ends, 6–8-septate, 35–52 × 13.5–22 µm ($\bar{x}\;$ = 42 × 18.5 µm, n = 30), smooth-walled, guttulate, truncate at the base, with an obconical basal cell. **Sexual morph:** Undetermined.

Culture characteristics: conidia germinating on PDA within 24 h and swollen germ tubes produced from both ends. Colonies growing on PDA reaching 5–10 mm in two weeks at 25 °C in dark, circular, with velvety, dark olivaceous brown mycelium on the surface; in reverse dark brown with filiform margin.

Material examined: China, Guizhou Province, Chishui City, Sidonggou Waterfall, 25°27.38′ N, 107°39.85′ E, on submerged decaying twig in a freshwater stream, 11 July 2019, J Yang, CS 40-2 (HKAS 112617, **holotype**); ex-type living culture GZCC 20-0512; additional sequence, SSU: MZ868768.

Notes: *Distoseptispora fusiformis* can be distinguished from other species in the genus by relatively long conidiophores with truncate apex and ellipsoidal to broadly fusiform, 6–8-septate, dark olivaceous brown to dark brown conidia with paler polar cells. *Distoseptispora fusiformis* was collected on the same material as *D. atroviridis* but they are distinct in morphology and phylogeny. *Distoseptispora atroviridis* can be distinguished from *D. fusiformis* by fasciculate or loosely compact conidiophores, trapezoidal conidiogenous cells and ellipsoidal to obovoid, dark green conidia. Conidiophores and conidia of *D. atroviridis* are longer and slightly smaller than those in *D. fusiformis* (100–167 µm vs. 40–110 µm; 31–43 × 13–20 µm vs. 35–52 × 13.5–22 µm), respectively. Comparing the LSU, ITS, *TEF1α*, and *RPB2* sequences of *D. atroviridis* and *D. fusiformis* showed 96.07% (32 bp differences), 90.21% (55 bp differences), 93.77% (56 bp differences), and 89.56% (114 bp differences) sequence similarity, respectively.

**Figure 6:**
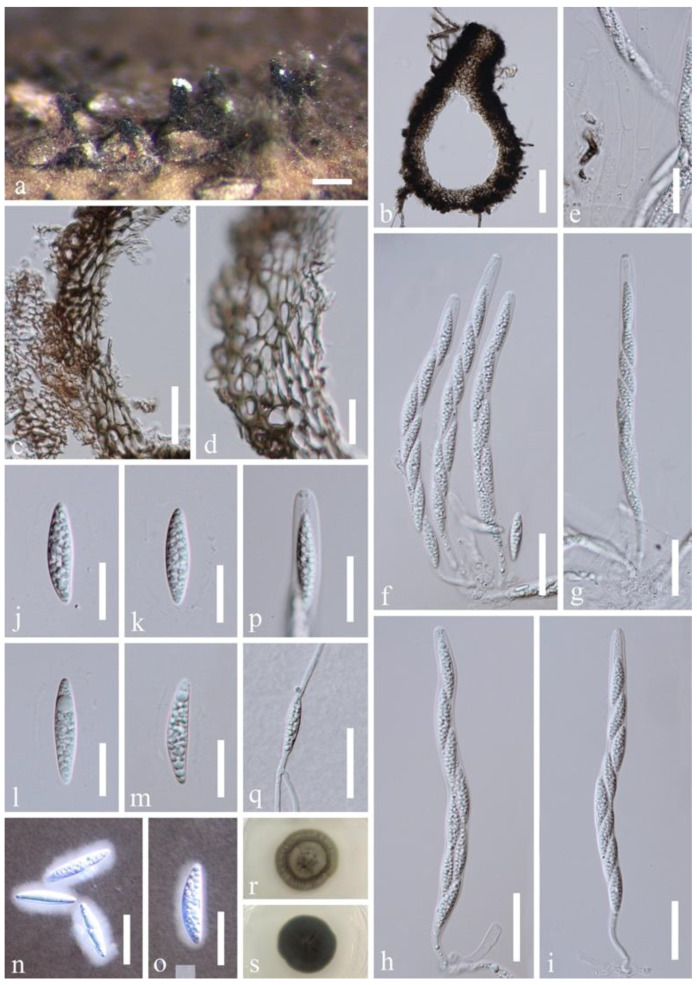
*Distoseptispora hyalina* (MFLU 21-0137, holotype). (**a**) Ascomata on woody substrate. (**b**) Vertical section of an ascoma. (**c**) Section of the bottom wall. (**d**) Section of the lateral wall near the beak. (**e**) Paraphyses. (**f**–**i**) Asci. (**j**–**o**) Ascospores, n and o using Indian ink. (**p**) Apical ring. (**q**) Germinated spore. (**r**,**s**) Culture, r from above, s from below. Scale bars: (**a**) 200 μm, (**b**) 50 μm, (**f**–**i**,**q**) 30 μm, (**c**,**e**,**n**,**p**) 20 μm, (**j**–**m**,**o**) 15 μm, (**d**) 10 μm.

*Distoseptispora hyalina* J. Yang and K.D. Hyde, sp. nov., Figure 6.

Index Fungorum number: IF558673; Facesoffungi number: FoF10249.

Etymology: referring to the hyaline conidia.

Holotype: MFLU 21-0137.

Saprobic on decaying submerged wood in a freshwater habitat. **Sexual morph:** Ascomata solitary or gregarious, immersed to semi-immersed, perithecial, 150–200 μm high, 120–190 μm diam., subglobose to ellipsoidal, dark brown, ostiolate, with a short neck erumpent through host surface. Ostiole periphysate. Ascomatal wall coriaceous, 20–31 μm thick, 2-layered; outer layer consisting of multi-rows of brown, thick-walled, polyhedral cells of textura angularis, inner layer comprising multi-rows of pale brown to hyaline, thin-walled, elongated cells of textura angularis or taxtura prismatica. Paraphyses sparse, persistent, septate, hyaline, tapering towards the apex, c. 4–7 μm wide near the base, constricted at the septa. Asci 145–190 × 8–11 µm ($\bar{x}$ = 165 × 9.8 µm, n = 20), cylindrical, with a short pedicel, obtuse at the apex, 8-spored, apex with a non-amyloid apical annulus. Ascospores (19.5–)23–26(–28.5) × 4.5–7 µm ($\bar{x}$ = 25 × 6 µm, n = 30), overlapping, uniseriate, fusiform, straight, rarely slightly curved, hyaline, 0–3-septate, smooth-walled, guttulate, thin-walled, with a mucilaginous sheath. **Asexual morph:** Undetermined.

Culture characteristics: conidia germinating on PDA within 24 h. Germ tubes produced from both ends. Colonies on PDA reaching 10–15 mm diam. after 2 weeks at 25 °C in natural light, with dense mycelium on the surface, gray in the middle, dark grayish green of the inner ring, and grayish green of the outer ring; in reverse dark olivaceous green with entire margin.

Material examined: Thailand, Trat Province, Amphoe Ko Chang, 12°7.98′ N, 102°37.98′ E, on decaying wood submerged in a freshwater stream, 27 April 2017, YZ Lu, YJT 26-1 (MFLU 21-0137, **holotype**); ex-type living culture MFLUCC 17-2128; additional sequence, SSU: MZ868765.

Notes: *Distoseptispora hyalina* is the first sexual morph reported in the genus based on molecular DNA data. *Distoseptispora hyalina* resembles *Sporidesmium thailandense* in possessing cylindrical, pedicellate asci with a non-amyloid apical annulus and obliquely uniseriate, fusiform, hyaline ascospores with a mucilaginous sheath. However, *D. hyalina* possesses immersed to semi-immersed, erumpent ascomata, larger brown cells of ascomatal wall and 3-septate ascospores when mature while S. *thailandense* has immersed ascomata with compact layers of cells of the peridium that is undifferentiated from host tissue and 3–4-septate ascospores [10,48]. In addition, *D. hyalina* differs from *S. thailandense* by the smaller asci (145–190 × 8–11 µm vs. 160–220 × 11–14 µm) and narrower ascospores but similar in length (4.5–7 µm wide vs. 8–10 µm wide). *Distoseptispora hyalina* is similar to *Miyoshiella triseptata* in having a non-amyloid apical annulus of asci and fusiform, 3-septate ascospores with comparable dimension (20–25 × 5–7 µm) [43], but *Miyoshiella triseptata* is distinguished by carbonaceous, papillate ascomata, shorter and broader asci (90–110 × 12–17 µm) and hyaline to light yellowish brown ascospores lacking a sheath [43].

In the phylogenetic analyses, *Distoseptispora hyalina* was sister to *D. yunnanensis* with good support (97% ML BS/1.0 PP/98% MP BS, Figure 1). The LSU, ITS, *TEF1α*, and *RPB2* sequence identity of *D. hyalina* and *D. yunnanensis* was 95.05% (807/849bp including 4bp of gaps), 94.59% (525/555bp including 9bp of gaps), 96.66% (898/929bp), and 93.41% (1035/1108bp), respectively.

*Distoseptispora hyalina* were colonized close to *D. amniculi* on the same twig in sexual and asexual stages, respectively. However, they are separate taxa based on molecular evidence.

*Distoseptispora lignicola* D.F. Bao, Z.L. Luo, H.Y. Su, and K.D. Hyde, Fungal Diversity 99: 487 (2019) Figure 7.

Index Fungorum number: IF555641, Facesoffungi number: FoF05413.

Cultural characteristics: conidia germinating on WA within 24 h and germ tube produced from both ends. Colonies on PDA reaching about 30 mm diam. after 3 weeks at 25 °C in dark, circular, with dense, velvety, whitish brown mycelium on the surface, uneven in the middle; in reverse dark brown at the entire margin, rimous in the middle.

Material examined: China, Guizhou Province, Chishui City, Chishui river basin, near 28°25′ N, 106°0′ E, at an altitude of 500 m, on submerged decaying wood in a freshwater stream, July 2019, LL Liu, CS 1-5-1 (GZAAS 20-0424), living culture GZCC 19-0529.

Notes: Our collection GZAAS 20-0424 matches the original diagnosis of the holotype of *Distoseptispora lignicola* (MFLU 18-1458) well [13]. Comparison of their LSU and ITS sequences showed 100% and 99.43% (526/529bp) similarity, respectively. We therefore identify our two collections as *D. lignicola* and report a new geographical record of this species in China.

**Figure 8:**
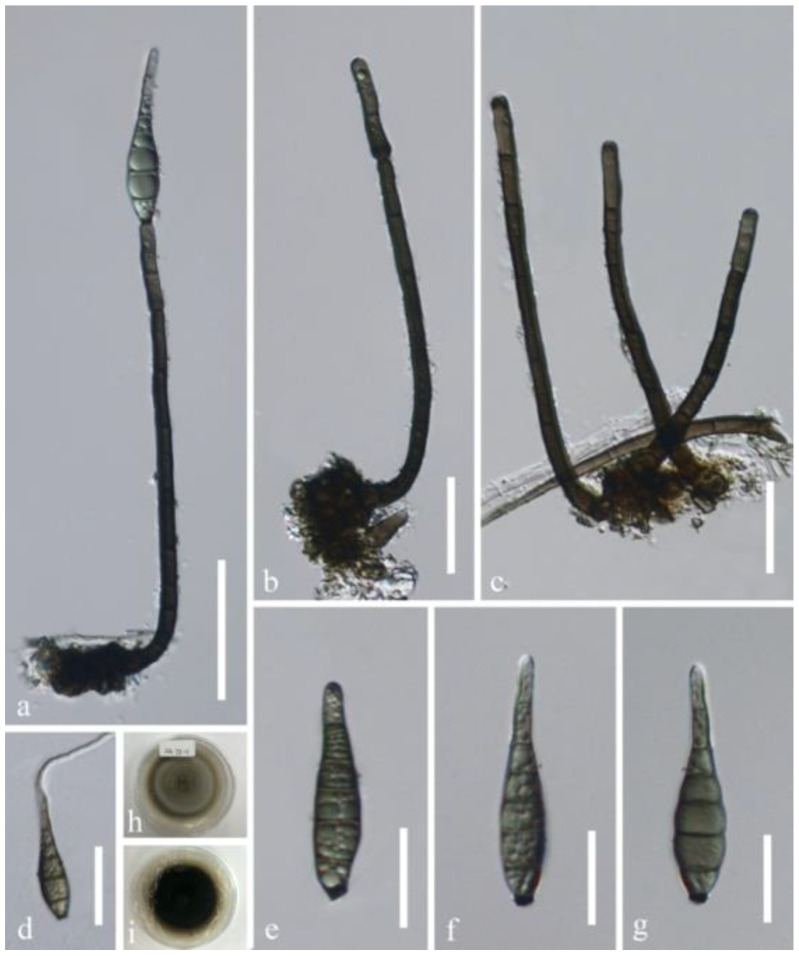
*Distoseptispora verrucosa* (HKAS 112652, holotype). (**a**) Conidiophore with a conidium. (**b**,**c**) Conidiophores. (**d**) Germinated conidium. (**e**–**g**) Conidia. (**h**,**i**) Culture, h from above, i from below. Scale bars: (**a**) 50 μm, (**b**–**d**) 30 μm, (**e**–**g**) 20 μm.

*Distoseptispora verrucosa* J. Yang and K.D. Hyde, sp. nov., Figure 8.

Index Fungorum number: IF558674; Facesoffungi number: FoF10251.

Etymology: referring to the verrucose conidia.

Holotype: HKAS 112652.

Saprobic on submerged decaying wood in a freshwater habitat. **Asexual morph:** Colonies on wood effuse, hairy, dark brown to black, scattered or in small groups, glistening. Mycelium partly immersed, partly superficial, composed of septate, smooth-walled, pale brown to hyaline hyphae. Conidiophores macronematous, mononematous, erect, solitary or caespitose, straight or flexuous, cylindrical, rounded and usually darkened at the apex, smooth-walled, septate, unbranched, dark brown, slightly paler at the upper part, 92–250 × 4.7–6.3 µm ($\bar{x}$ = 162 × 5.7 µm, n = 20). Conidiogenous cells monoblastic, integrated, terminal, determinate, sometimes percurrently proliferating, cylindrical, brown. Conidia acrogenous, solitary, obclavate, rostrate, upper part tapering towards the apex, olivaceous brown, becoming paler at the apex, 6–8-septate, 41–63 × 8.8–12.6 µm ($\bar{x}$ = 51.5 × 10.8 µm, n = 30), verrucose, guttulate, truncate with a darkened scar at the base. **Sexual morph:** Undetermined.

Cultural characteristics: conidia germinating on PDA within 24 h and germ tubes produced from both ends. Colonies on PDA reaching 5–10 mm diam. after 14 days at 25 °C, in natural light, circular, with velvety, dense, grayish brown mycelium on the surface with entire margin; in reverse dark brown.

Material examined: China, Guizhou Province, Dushan District, 25°57.9′ N, 107°39′ E, on decaying wood submerged in a freshwater stream, 26 Aug 2017, J Yang, SG 75-1 (HKAS 112652, **holotype**), ex-type living culture GZCC 20-0434; additional sequence, SSU: MZ868767.

Notes: In the phylogenetic analyses, *Distoseptispora verrucosa* clustered with *D. lancangjiangensis*, *D. suoluoensis*, and *D. bambusae*. Their systematic placement is correlated to the highly similar morphological characters in having dark brown conidiophores with rounded apex and narrowly obclavate, rostrate, euseptate, verrucose conidia with a dark scar at the base. They differ in conidial color and dimensions: *D. verrucosa* has the smallest, olivaceous brown conidia 41–63 × 8.8–12.6 µm; *D. suoluoensis* [10] has the largest, dark yellowish brown to dark olivaceous brown conidia (65–)80–125(–145) × 8–13 μm usually with a secondary conidium; *D. bambusae* [23] and *D. lancangjiangensis* [44] possess brown or olivaceous conidia measuring 45–74 × 5.5–9.5 µm and 64–84 × 9–10 μm, respectively. The length of conidiophores of *D. verrucosa* (92–250 µm) and *D. suoluoensis* (80–250 µm) are comparable and longer than that in *D. bambusae* (40–96 µm) and *D. lancangjiangensis* (144–204 µm). Phylogenetically, *D. verrucosa* was sister to *D. suoluoensis*, showing 99.88% (838/839 bp), 98.65% (513/520 bp), 98.44% (884/898 bp), and 98.25% (1015/1033 bp) sequence identity of LSU, ITS, *TEF1α*, and *RPB2* sequences, respectively. *Distoseptispora verrucosa* resembles *Sporidesmium tengii* and *S. tunicatum* with long conidiophores and obclavate, rostrate, euseptate conidia. However, conidiophores in the latter two species are truncate at the apex and conidia lack a basal darkened scar. *Sporidesmium tengii* [6] differs by shorter conidiophores (60–100 µm) and smooth-walled, smaller conidia (45–50 × 7–8.5 µm). *Sporidesmium tunicatum* [49] has shorter conidiophores (110–180 µm), verrucose but slightly longer conidia (43–75 × 9–13 µm) with an apical sheath. Molecular DNA data are not available for *S. tengii* and *S. tunicatum*.

## 5. Discussion

The establishment of *Distoseptispora* [9] was based on morphology and molecular DNA data. More than 30 species in the genus are supported by sequence data. The genus forms monophyletic clade (Figure 1) distinct from other sporidesmium-like taxa. Members in the genus mainly occur in the asexual morph, forming effuse, hairy colonies on decaying wood, bamboo culms, plant stems, rachis, and fallen leaves from terrestrial and freshwater habitats. The morphological concept of *Distoseptispora* is macronematous, mononematous, solitary or fasciculate conidiophores, sometimes elongating percurrently and rarely reduced to conidiogenous cells; monoblastic or polyblastic, cylindrical or clavate conidiogenous cells; conidia are cylindrical, obclavate, rostrate, ellipsoidal, obovoid or fusiform, subhyaline, olivaceous, dark green, brown or yellowish-brown to reddish-brown, euseptate or distoseptate, rarely muriform, sometimes born a secondary conidium, with or without septal pore and mucilaginous sheath. The characters delineating the genus *Distoseptispora* also cover the criteria of *Ellisembia* and *Sporidesmium*. *Ellisembia* was segregated from the widely circumscribed *Sporidesmium* by Subramanian [1] based on the septal type. However, given the non-taxonomic value of euseptate and distoseptate conidia among sporidesmium-like species, Su et al. [9] recognized the distoseptate *Ellisembia* as a synonym of the redefined *Sporidesmium sensu stricto* which forms a robust monoclade and accommodates species with distoseptate/euseptate, obclavate or subcylindrical conidia, and conidiophores with or without percurrent extensions. In this study, we follow Hyde et al. [12] in treating them as separate taxa because of their unclear relationship due to the absence of molecular DNA data from their type species.

Several sexual morphs have been linked to *Ellisembia* and *Sporidesmium* through cultural and/or molecular studies. *Ellisembia folliculata* (sexual morph: *Lecythothecium duriligni*) [50] and *E. aurea* [12] differ from the sexual morph *D. hyalina* by versicolorous ascospores and position within *Chaetosphaeriaceae* (*Chaetosphaeriales*, *Sordariomycetes*). *Sporidesmium thailandense* [10,48] and *S. lignicola* [13] can be distinguished by brown ascomata with a hyaline neck and compact, elongated cells of the ascomatal wall, outer layer undifferentiated from host tissue, and their systematic placement in *Sporidesmiaceae* (*Sporidesmiales*, *Sordariomycetes*). The morphology of sexual morphs of sporidesmium-like genera along with molecular DNA data characterizes their identification although they have similar asexual morphs.

It is challenging to identify some *Distoseptispora* species with highly similar morphology, such as those of *D. multiseptata*, *D. phangngaensis*, and *D. xishuangbannaensis*. Still, they can be well separated by molecular DNA data [10,16,18]. Some *Distoseptispora* species have a wide range of conidial length, for example, conidia of *D. multiseptata* are 95–290 µm long of the holotype but 300–700 µm long in the additional collection; those in *D. phangngaensis* 165–350 µm long and *D. tectonigena* 83–360 µm long [9,10,16]. The indeterminate conidial length may depend on the incubation period. Thus, conidial length is less taxonomically informative in separating some *Distoseptispora* species. The type of septa, however, is proven to have no taxonomic significance for generic delimitation of sporidesmium-like taxa, but it is informative at the species level [10,14].

Su et al. [9] accepted two former *Ellisembia* species *E. adscendens* and *E. leonensis* in *Distoseptispora* that were not validly published, based on the non-type strains “*Distoseptispora adscendens*” HKUCC 10820 and “*D. leonensis*” HKUCC 10822 lacking associated morphology. *Ellisembia adscendens* was initially introduced as *Sporidesmium adscendens* forming elongated black patches on the pileus of *Polyporus versicolor* Fr. (No. 1345) which was collected on the underside of timber in the Falkland Islands [51]. *Ellisembia adscendens* is a widespread species. It is similar to *E. vaga* [52] but differs by wider conidia. *Ellisembia adscendens* and *E. vaga* highly resemble a group of morphologically indistinguishable species in *Distoseptispora*, e.g., *D. multiseptata* and *D. phangngaensis*. They are probably members of *Distoseptispora* because of the typical *Distoseptispora* morphology in having short conidiophores and subcylindrical long conidia. On the other hand, the large number of specimens of *Ellisembia adscendens* may be a complex comprising several separate taxa. *Ellisembia leonensis* [47] is characterized by relatively long conidiophores with percurrent extensions and fusiform to rostrate, distoseptate conidia. It matches the morphological concept of both *Distoseptispora* and *Sporidesmium sensu stricto*. At present, we avoid reassigning *E. adscendens* and *E. leonensis* in *Distoseptispora* until their systematic placement is confirmed by molecular data from type materials or resolved by epitypifications.

## Figures and Tables

**Figure 7:**
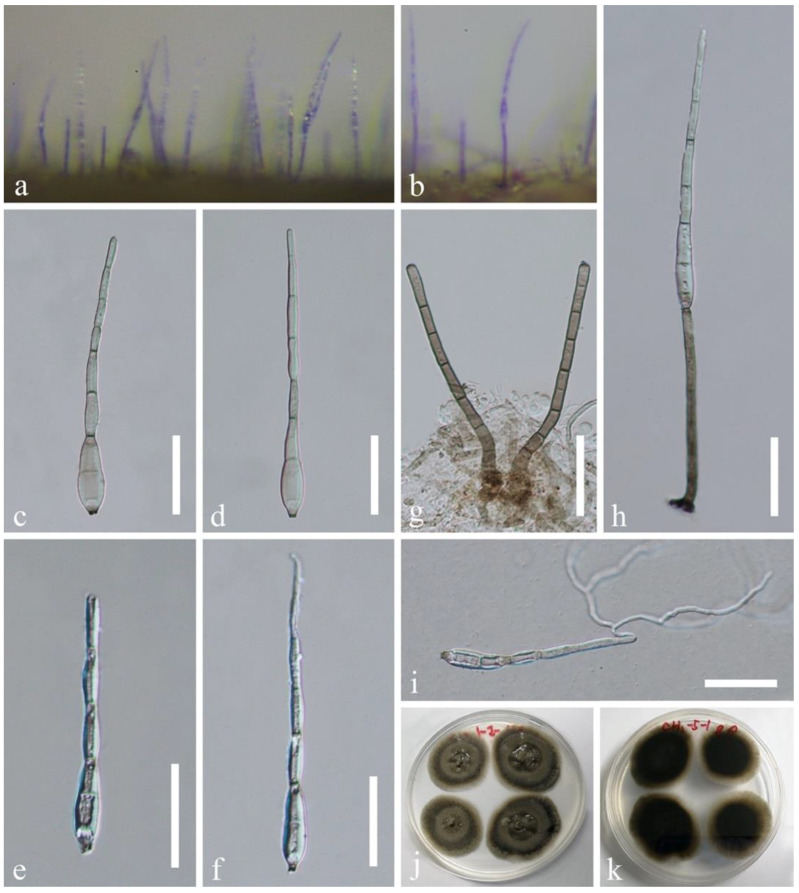
*Distoseptispora lignicola* (GZAAS20-0424). (**a**,**b**) Colonies on natural substrate. (**c**–**f**) Conidia. (**g**) Conidiophores. (**h**) Conidiophore and a conidium. (**i**) Germinated conidium. (**j**,**k**) Culture, j from above, k from below. Scale bars: (**c**–**i**) 30 μm.

**Table 1:** Taxa used in the phylogenetic analyses and their GenBank accession numbers. T denotes ex-type strains. Newly generated sequences are in bold.

Taxon	Voucher/Strain Number	GenBank Accession Number			
		LSU	ITS	*TEF1α*	*RPB2*
*Acrodictys bambusicola*	CGMCC 3.18641	KX033564	KU999973	–	–
*Acrodictys elaeidicola*	CGMCC 3.18642	KX033569	KU999978	–	–
*Aquapteridospora aquatica*	MFLUCC 17-2371^T^	MW287767	MW286493	–	–
*Aquapteridospora fusiformis*	MFLUCC 18-1606^T^	MK849798	MK828652	MN194056	–
***Aquapteridospora lignicola***	**MFLUCC 15-0377** ^T^	KU221018	**MZ868774**	**MZ892980**	**MZ892986**
*Bullimyces aurisporus*	AF316-1b^T^	JF775590	–	–	–
*Bullimyces communis*	AF281-5	JF775587	–	–	–
*Cancellidium applanatum*	CBS 337.76^T^	MH872755	MH860985	–	–
*Cancellidium cinereum*	MFLUCC 18-0424^T^	MT370363	MT370353	MT370488	MT370486
“*Distoseptispora adscendens*”	HKUCC 10820	DQ408561	–	–	DQ435092
***Distoseptispora amniculi***	**MFLUCC 17-2129** ^T^	**MZ868761**	**MZ868770**	–	**MZ892982**
*Distoseptispora appendiculata*	MFLUCC 18-0259^T^	MN163023	MN163009	MN174866	–
*Distoseptispora aquatica*	MFLUCC 15-0374^T^	KU376268	MF077552	–	–
*Distoseptispora aquatica*	MFLUCC 18-0646	MK849793	MK828648	MN194052	–
*Distoseptispora aquatica*	S-965	MK849792	MK828647	MN194051	MN124537
***Distoseptispora atroviridis***	**GZCC 20-0511** ^T^	**MZ868763**	**MZ868772**	**MZ892978**	**MZ892984**
***Distoseptispora atroviridis***	**GZCC 19-0531**	**MZ227223**	**MW133915**	–	–
*Distoseptispora bambusae*	MFLUCC 20-0091^T^	MT232718	MT232713	MT232880	MT232881
*Distoseptispora bambusae*	MFLUCC 14-0583	MT232717	MT232712	–	MT232882
*Distoseptispora bangkokensis*	MFLUCC 18-0262^T^	MZ518206	MZ518205	–	–
*Distoseptispora cangshanensis*	MFLUCC 16-0970^T^	MG979761	MG979754	MG988419	–
*Distoseptispora caricis*	CPC 36498^T^	MN567632	MN562124	–	MN556805
*Distoseptispora caricis*	CPC 36442^T^	–	MN562125	–	MN556806
*Distoseptispora chinensis*	GZCC 21-0665^T^	MZ474867	MZ474871	MZ501609	–
*Distoseptispora clematidis*	MFLUCC 17-2145^T^	MT214617	MT310661	–	MT394721
*Distoseptispora dehongensis*	KUMCC 18-0090T	MK079662	MK085061	MK087659	–
***Distoseptispora effusa***	**GZCC 19-0532** ^T^	**MZ227224**	**MW133916**	**MZ206156**	–
*Distoseptispora euseptata*	MFLUCC 20-0154^T^	MW081544	MW081539	–	MW151860
*Distoseptispora euseptata*	DLUCC S2024	MW081545	MW081540	MW084994	MW084996
*Distoseptispora fasciculata*	KUMCC 19-0081^T^	MW287775	MW286501	MW396656	–
*Distoseptispora fluminicola*	MFLUCC 15-0417^T^	KU376270	MF077553	–	–
***Distoseptispora fusiformis***	**GZCC 20-0512** ^T^	**MZ868764**	**MZ868773**	**MZ892979**	**MZ892985**
*Distoseptispora guizhouensis*	GZCC 21-0666^T^	MZ474869	MZ474868	MZ501610	MZ501611
*Distoseptispora guttulata*	MFLUCC 16-0183^T^	MF077554	MF077543	MF135651	–
***Distoseptispora hyalina***	**MFLUCC 17-2128** ^T^	**MZ868760**	**MZ868769**	**MZ892976**	**MZ892981**
*Distoseptispora hydei*	MFLUCC 20-0115^T^	MT742830	MT734661	–	MT767128
*Distoseptispora lancangjiangensis*	DLUCC 1864^T^	MW879522	MW723055	–	–
“*Distoseptispora leonensis*”	HKUCC 10822	DQ408566	–	–	DQ435089
*Distoseptispora lignicola*	MFLUCC 18-0198^T^	MK849797	MK828651	–	–
***Distoseptispora lignicola***	**GZCC 19-0529**	**MZ227219**	**MW133911**	–	–
*Distoseptispora longispora*	HFJAU 0705^T^	MH555357	MH555359	–	–
*Distoseptispora martinii*	CGMCC 3.18651	KX033566	KU999975	–	–
*Distoseptispora multiseptata*	MFLUCC 15-0609^T^	KX710140	KX710145	MF135659	–
*Distoseptispora multiseptata*	MFLUCC 16-1044	MF077555	MF077544	MF135652	MF135644
*Distoseptispora neorostrata*	MFLUCC 18-0376^T^	MN163017	MN163008	–	–
*Distoseptispora obclavata*	MFLUCC 18-0329^T^	MN163010	MN163012	–	–
*Distoseptispora obpyriformis*	MFLUCC 17-1694^T^	MG979764	–	MG988422	MG988415
*Distoseptispora obpyriformis*	DLUCC 0867	MG979765	MG979757	MG988423	MG988416
*Distoseptispora palmarum*	MFLUCC 18-1446^T^	MK079663	MK085062	MK087660	MK087670
*Distoseptispora phangngaensis*	MFLUCC 16-0857^T^	MF077556	MF077545	MF135653	–
*Distoseptispora rayongensis*	MFLUCC 18-0415^T^	MH457137	MH457172	MH463253	MH463255
*Distoseptispora rayongensis*	MFLUCC 18-0417	MH457138	MH457173	MH463254	MH463256
*Distoseptispora rostrata*	MFLUCC 16-0969^T^	MG979766	MG979758	MG988424	MG988417
*Distoseptispora rostrata*	DLUCC 0885	MG979767	MG979759	MG988425	–
*Distoseptispora saprophytica*	MFLUCC 18-1238^T^	MW287780	MW286506	MW396651	MW504069
*Distoseptispora songkhlaensis*	MFLUCC 18-1234^T^	MW287755	MW286482	MW396642	–
*Distoseptispora suoluoensis*	MFLUCC 17-0224^T^	MF077557	MF077546	MF135654	–
***Distoseptispora suoluoensis***	**MFLUCC 17-1305**	MF077558	MF077547	–	**MZ945510**
*Distoseptispora tectonae*	MFLUCC 12-0291^T^	KX751713	KX751711	KX751710	KX751708
*Distoseptispora tectonae*	MFLUCC 15-0981	MW287763	MW286489	MW396641	–
*Distoseptispora tectonae*	MFLUCC 16-0946	MG979768	MG979760	MG988426	MG988418
*Distoseptispora tectonigena*	MFLUCC 12-0292^T^	KX751714	KX751712	–	KX751709
*Distoseptispora thailandica*	MFLUCC 16-0270^T^	MH260292	MH275060	MH412767	–
*Distoseptispora thysanolaenae*	KUMCC 18-0182^T^	MK064091	MK045851	MK086031	–
*Distoseptispora thysanolaenae*	HKAS 112710	MW879524	MW723057	MW729783	–
***Distoseptispora verrucosa***	**GZCC 20-0434** ^T^	**MZ868762**	**MZ868771**	**MZ892977**	**MZ892983**
*Distoseptispora xishuangbannaensis*	KUMCC 17-0290^T^	MH260293	MH275061	MH412768	MH412754
*Distoseptispora yunnanensis*	MFLUCC 20-0153^T^	MW081546	MW081541	MW084995	MW151861
*Fluminicola saprophytica*	MFLUCC 15-0976^T^	MF374367	MF374358	MF370956	MF370954
*Myrmecridium banksiae*	CPC 19852^T^	JX069855	JX069871	–	–
*Myrmecridium schulzeri*	CBS 100.54	EU041826	EU041769	–	–
*Papulosa amerospora*	AFTOL-ID 748	DQ470950	–	DQ471069	DQ470901
*Pleurophragmium bambusinum*	MFLUCC 12-0850	KU863149	KU940161	KU940213	–
*Pseudostanjehughesia aquitropica*	MFLUCC 16-0569^T^	MF077559	MF077548	MF135655	–
*Pseudostanjehughesia lignicola*	MFLUCC 15-0352^T^	MK849787	MK828643	MN194047	MN124534
*Wongia griffinii*	DAR 80512^T^	KU850471	KU850473	–	–

## Data Availability

The datasets generated for this study can be found in the NCBI database.
